# Top considerations for creating bioinformatics software documentation

**DOI:** 10.1093/bib/bbw134

**Published:** 2017-01-14

**Authors:** Mehran Karimzadeh, Michael M Hoffman

**Affiliations:** Department of Medical Biophysics, University of Toronto, Toronto, Canada

**Keywords:** software, documentation, perspective

## Abstract

Investing in documenting your bioinformatics software well can increase its impact and save your time. To maximize the effectiveness of your documentation, we suggest following a few guidelines we propose here. We recommend providing multiple avenues for users to use your research software, including a navigable HTML interface with a quick start, useful help messages with detailed explanation and thorough examples for each feature of your software. By following these guidelines, you can assure that your hard work maximally benefits yourself and others.

## Introduction

You have written a new software package far superior to any existing method. You submit a paper describing it to a prestigious journal, but it is rejected after Reviewer 3 complains they cannot get it to work. Eventually, a less exacting journal publishes the paper, but you never get as many citations as you expected. Meanwhile, there is not even a single day when you are not inundated by emails asking very simple questions about using your software. Your years of work on this method have not only failed to reap the dividends you expected, but have become an active irritation. And you could have avoided all of this by writing effective documentation in the first place.

Academic bioinformatics curricula rarely train students in documentation. Many bioinformatics software packages lack sufficient documentation. Developers often prefer spending their time elsewhere. In practice, this time is often borrowed, and by ducking work to document their software now, developers accumulate ‘documentation debt’. Later, they must pay off this debt, spending even more time answering user questions than they might have by creating good documentation in the first place. Of course, when confronted with inadequate documentation, some users will simply give up, reducing the impact of the developer’s work.

To avoid this, we suggest several guidelines for improving multiple aspects of your documentation (Table 1). These guidelines improve the usability of your software and reduce time spent supporting users. Many of these guidelines apply both to bioinformatics software and to bioinformatics databases. In this perspective, we describe in detail the best practices of many well-established bioinformatics tools (Table 2).

**Table 1:** A Taxonomy of research software documentation

Format	Content	Audience
Manuscript	Conceptual and technical details of the method	New users
Readme	Basic instructions for installation and use of the software and where to find more information	New users
Quick start	Step-by-step instructions for installation and use of the software on a provided test data set	New users
Reference manual	Complete details of every configurable setting, input and output	All users
FAQ	Answers to commonly asked or anticipated questions	All users
Searchable forum or mailing list	News and discussion of details not otherwise provided in the documentation or not apparent to users	All users
Built-in help	Concise description of a software component and its parameters	Experienced users
News	Changes in behavior, bug fixes, new features and caveats	Experienced users
Code comments	Extensive details of implementation	Power users

**Table 2:** Documentation formats provided by selected bioinformatics software packages

	Cites	MS	Rea	QS	Ref	FAQ	For	Hel	New	Com
BLAST [1]	61,534	+	+	+	+	+	+	+	+	+
MEGA [2]	28,153	+	−	+	+	+	−	+	+	−
PLINK [3]	10,935	+	+	+	+	+	+	+	+	+
Swiss-PdbViewer [4]	9,470	+	−	−	+	−	+	−	+	−
SAMtools [5]	9,176	+	+	−	+	+	+	+	+	+
BWA [6]	8,963	+	+	−	+	+	+	+	+	+
EMBOSS [7]	4,898	+	+	−	+	+	+	+	+	+
Bowtie [8]	4,397	+	−	+	+	+	+	+	+	+
DESeq [9]	4,271	+	−	−	+	−	−	+	+	+
Cufflinks [10]	4,166	+	+	+	+	−	+	+	+	+
GATK [11]	4,146	+	−	+	+	+	+	+	+	+
limma [12]	3,714	+	−	+	+	−	−	+	−	+
edgeR [13]	3,671	+	−	+	+	−	−	+	−	+
MACS [14]	2,824	+	+	−	+	+	+	+	+	+
Bedtools [15]	2,746	+	+	+	+	+	+	+	+	+
Clustal Omega [16]	2,465	+	+	−	−	−	−	+	+	+
Meme Suite [17]	1,889	+	+	+	+	+	+	+	+	+
Trimmomatic [18]	1,449	+	−	+	+	−	−	+	+	+
STAR [19]	1,080	+	+	−	+	−	+	+	+	+
Segway [20]	209	+	+	+	+	+	+	+	+	+
Bioconductor [21]	157	+	+	+	+	+	+	+	+	+
Picard Tools [22]	NA	−	+	+	+	+	+	+	−	+

Cites, number of citations on 29 August 2016 (Google Scholar); MS, peer-reviewed manuscript; Rea, readme; QS, quick start; Ref, reference manual; FAQ, frequently asked questions; For, forum or mailing list; Hel, built-in help; New, news; Com, code comments.

## Guidelines for great documentation

### Hierarchical documentation

Your documentation should consist in hierarchically grouped and carefully sorted components. This allows users to efficiently find the detail they need without overwhelming them with a large span of top-level material. It limits the amount of information shown to the user at one time, and it sorts the most important materials at the top and less frequently used details at the bottom.

**Figure 1:**
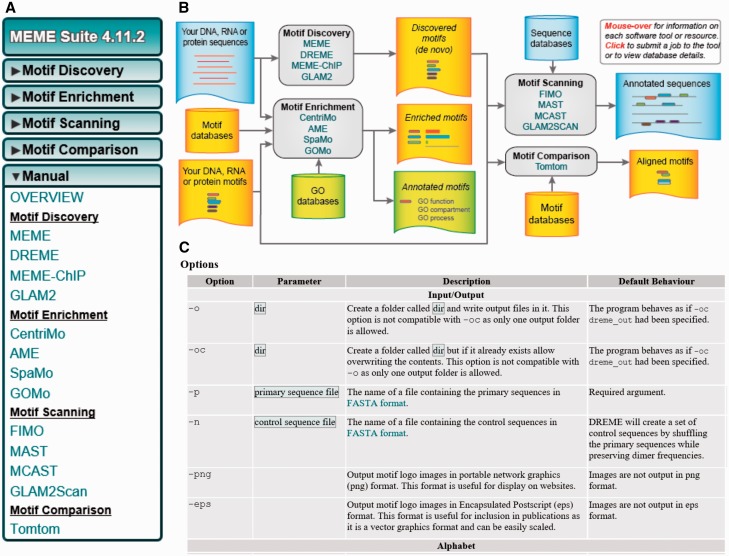
Multilevel hierarchy in the MEME Suite documentation. The MEME Suite provides a variety of tools for motif enrichment analysis. (**A**) The ‘Manual’ tab in the sidebar organizes individual tools into broad categories. Each tool then has a link to its own detailed reference manual. (**B**) The web site’s main page describes application of different tools in a flow chart, providing the context of how they work together. (**C**) A section of the DREME tool’s reference manual, showing further hierarchy and comprehensive detail. A four-column table describes details of each option in the DREME program. Each row describes a single option, and these options are categorized into broader option groups.

The MEME Suite contains multiple programs for sequence motif analysis. Its documentation begins with a flow chart that describes its modules and their relationship to each other (Figure 1B). Other top-level items provide information on installation, databases that the programs rely on, and ways to get support. The MEME Suite also has a top-level menu that groups programs by function (Figure 1A). More commonly used modules appear first. This grouping and ordering makes it easier for users to find the module they need and to compare with related tools for their task.

For example, the ‘Manual’ section of the sidebar, groups the programs into four categories—‘Motif Discovery’, ‘Motif Enrichment’, ‘Motif Scanning’ and ‘Motif Comparison’ (Figure 1A). The manual of each program within describes both the web and command-line interfaces. As an illustrative sub-example, we will examine further the manual for DREME, one of the MEME Suite’s motif discovery tools. Its command-line documentation consists in several components. ‘Usage’ describes the minimal parameters for using the program. ‘Description’ includes a technical but abstract explanation of DREME’s functionality. The manual comprehensively defines ‘Input’ and ‘Output’ formats and describes options in detail using a table (Figure 1C). This table groups the options in several categories such as ‘Input/Output’, ‘Alphabet’, ‘General’, and ‘Miscellaneous’. For each option, this table describes the parameters, description and the default behavior in subsequent columns. The MEME Suite concludes each program’s manual with a citation to the peer-reviewed manuscript describing that program.

**Figure 2:**
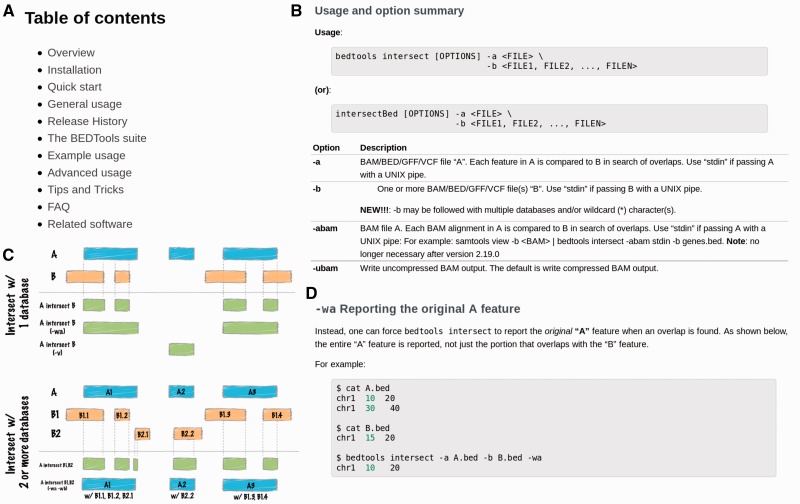
Hierarchy, extensive examples and visual diagrams in the Bedtools documentation. (**A**) The Bedtools documentation uses a well-organized hierarchy to provide appropriate entry points for new or experienced users. (**B**) Bedtools breaks its documentation into an individual page for every sub-command, describing every parameter of every module in detail and documenting changes in different versions. (**C**) A visual diagram demonstrates the effects of various options. (**D**) Bedtools demonstrates examples for every option.

Bedtools [15] provides another example of well-documented and widely used bioinformatics software. Bedtools has a table of contents that directs users to the information they need (Figure 2A). These contents consist in a hierarchy of information structured and stored for optimal retrieval (Figure 2). Bedtools notably uses informative figures and extensive examples to clarify the functionality of different options (Figure 2C).

### Tools for documentation

Several software packages automatically generate up-to-date documentation from a markup language in the source code and elsewhere. These tools transform your code and markup into formats such as Unix manual (‘man’) page, Hypertext Markup Language (HTML) and Portable Document Format (PDF). Ideally you will create all these formats, but we consider an HTML manual most essential.

Examples of documentation generators include Doxygen [23] and Sphinx [24]. Sphinx has particular popularity in bioinformatics owing to its use of the intuitive markup language reStructuredText [25] and extensive formatting options. Some tools generate documentation specifically for one programming language, such as Javadoc [26] for Java, or Roxygen [27] for R.

The main disadvantage of automatically generated documentation is that you have less control of how to organize the documentation effectively. Whether you used a documentation generator or not, however, there are several advantages to an HTML web site compared with a PDF document. Search engines will more reliably index HTML web pages. In addition, users can more easily navigate the structure of a web page, jumping directly to the information they need.

### Quick start

Design your manual with a ‘quick start’ that tells users exactly how to get a result with a small number of explicit steps on a specified test data set. If this data set is not included in your package, ensure one can download the data set quickly. The user should be able to follow your script exactly and get the same results you describe. Ensure that these steps are executed quickly.

For example, Segway [20] includes a quick start guide with four subsections covering installation and configuration, acquiring data, running Segway and results. Segway includes the data set for testing the software in its own repository.

### Graphical interfaces

Software with a graphical interface, such as web applications, also requires more graphical documentation. Describing how to interact with a graphical interface in text can prove laborious, and a well-annotated picture can be worth a hundred words. As an example, Swiss-PdbViewer [4] is graphical software that models protein structure. Its documentation makes ample use of screenshots and visuals that depict elements of the Swiss-PdbViewer interface, such as icons. These visuals help users to quickly understand how to complete tasks, and to interpret the software’s output.

### Installation

Describe how to install your software and all of its dependencies, in detail. At a minimum, provide exact instructions for the most recent versions of Debian, Red Hat Enterprise Linux, macOS and Windows—or the subset of those systems that you support. It is laborious to support multiple versions of an operating system, but that does not excuse avoiding these instructions for at least one version. Indicate a known working version of all of the dependencies, as well. Many scientists use computing clusters or network computers where they lack root privileges. When possible, your instructions should cover root and non-root installation.

Ensure you test installation on a new, unconfigured environment. A continuous integration service (see below) provides a great means for accomplishing this. If you use non-standard build tools or your software has complex dependencies, document the installation thoroughly and extensively. Sometimes it is easier for you to make installation easier for users. If your installation instructions seem complex, consider ways to make it easier, perhaps by contributing your software to a package repository such as Debian Med [28], Homebrew [29] or the Comprehensive R Archive Network [30].

PLINK [3] provides a good example of bioinformatics software supporting all major operating systems, with detailed instructions for each platform.

### Readme and news

Provide a readme file at the top level of your source code with basic information about installation and use of your software, and details on where users can find more information. The readme should show up to users visiting your source code repository and will provide the first impression for many. The readme should also include the software’s license.

Also, provide a news section dedicated to the changes in each release of the software. Discuss bug fixes, caveats, new features and changes in behavior of the software in detail. Users will often upgrade after several new versions, and want a place to find the details of all that has changed since their last install. Include the news as another file in the top level of your source code and link to it from the readme.

### File formats

If you must create a new file format (and please do not, if you can avoid it), make sure to specify it in detail. Burying specification details in your code make operation with future software by others frustrating. A detailed specification, however, makes it easier to use your software in a larger pipeline, and reduces the chance you will have to debug interoperability problems later. The MEME Suite [17] and PLINK [31] both exemplify detailed description of input and output formats.

### Communication with users

Users may need to contact you if they cannot find the answers they need in the documentation. Set up a mailing list to allow users to send questions and feedback. Archive the mailing list where search engines can find it. People who encounter an error will report the message, allowing others to easily find the solution. Mailing lists facilitate an open development process, which may lead to users developing and submitting new features for your software. Some bioinformatics software packages, such as GATK [11], also host a forum which serves a similar purpose in making answers available to all. Forums, however, perform more poorly than mailing lists in getting others to contribute. New submissions to mailing lists are pushed to all list members, including those who registered to ask their own questions or learn about software updates. In forums, however, users must actively check the forum to see new questions. Often only the developers have the motivation to do this.

Issue trackers provide a great way to communicate about specific potential bugs or requests. GitHub [32] and Bitbucket [33] provide a free service for issue tracking, along with a repository for your code and documentation.

Adding a comment section to your documentation, web page encourages users to contribute helpful feedback. So does Read the Docs [34], which makes it easy for users to submit a pull request correcting the documentation. If you receive repeated inquiries on one aspect of your software, this is evidence for insufficient documentation. Take this as a sign to revise the documentation.

MISO [35], ggplot2 [36] and Bedtools [15] provide detailed documentation in HTML format, have a public GitHub repository to track issues, and also have a mailing list for other communications with users.

### Frequently asked questions

Prepare a frequently asked questions (FAQ) document to answer common questions you expect or have received. Many users find the FAQ format more compelling than a reference manual, and it is easier to link to an answer to a common question from a mailing list. PLINK has an FAQ that covers a variety of difficulties one may encounter before starting to use the software. It also includes questions that are related to unexpected outputs, and comparison with other packages.

### Troubleshooting

Your software should provide meaningful warning and error messages when it receives unexpected input. Include a chapter in your documentation to thoroughly explain error and warning messages and how to resolve them. When the users search the Internet for the text of these errors and warnings, they will find answers immediately.

## Technical choices and software documentation

### Programming environment

Using programming environments and languages that require difficult installation and configuration reduces the usability of your program, and they also require more complex documentation. For example, to run MATLAB programs without an expensive license, user must install a specific version of the MATLAB Compiler Runtime (MCR). Documenting all the things that can go wrong in installing an old version of MCR provides quite a challenge. This explains partially why few widely used bioinformatics tools rely on MATLAB.

### Default parameters

Many users rely on your default parameters, so choose them carefully. Configuration options left to potentially inexpert users provide no substitute for sensible defaults. Document the rationale for selecting any default parameter. This will help users understand when they should change it.

### Citation

Provide a citation to your own manuscript with a link to an open-access version. This makes it easier for users to find a description of your methodology and cite your work.

### Writing code

At some point, the documentation will not answer every question. At this point, someone must examine the source code and make it easy for that someone else to figure things out without help. That someone, invariably, will end up being yourself sometimes.

Put a premium on making your code easily intelligible to others. Use descriptive variable and function names following the standard format for your environment. PEP 8 [37] supplies a format for Python, and Google style guides [38] provide them for other programming languages. Many text editors can check code style automatically.

Comments provide an important avenue to increase code accessibility. Use a template to begin the header of your code with a comment including your name, email address and date of creation. At the top of each source code file, provide a brief description of its function. Concisely annotate your code with block or inline comments whenever it does anything not understood with trivial effort. If you use a documentation generator, use specially formatted comments to annotate functions with structured information.

### Continuous integration of quick start and tests

Your quick start effectively provides a simple script on a small test data set. Not only does this familiarize users with features of your software, but it also ensures that the software is installed properly and functions as expected.

You or other contributors can also use this script as a quick test to ensure that changes do not break any part of the software, or your instructions. You should therefore include the major options of your software in this script.

Consistent version control with Git or Mercurial helps you and collaborators track the development of the project and contribute easily. Using tools for coverage or mutation test of your code and continuous integration services such as drone.io [39], which supports both GitHub and Bitbucket, help you identify potential problems with your program faster.

## Discussion

While many bioinformatics software packages have satisfactory documentation, insufficient documentation makes others unusable by the community. Well-documented software is also an important aspect of reproducible analysis [40, 41]. Several previous reviews include checklists for bioinformatics software engineering that include software documentation [42–44]. Despite this, many bioinformatics software developers do not prioritize the creation of documentation. Nguyen-Hoan et al. [45] performed a survey asking 60 scientific software developers about their development practices. While 51 of 60 participants used inline code comments, fewer supplied the other documentation formats such as installation instructions (42 of 60) or user manuals (30 of 60) suggested here. Clearly, there is a long way to go in educating bioinformatics software developers on the best practices of effective documentation.

Although documentation is often mentioned as an important element of bioinformatics software engineering, little primary research specifically focuses on bioinformatics documentation. One can find primary research, however, on the effects of software documentation more generally. Junji et al. [46] reviewed the literature on software documentation research, and quantified how often documentation was shown to improve various aspects of software engineering. Documentation is shown to have a positive influence on software maintenance (29 articles), software development (16 articles), code comprehension (14 articles) and software design comprehension (10 articles). One study shows that initial documentation improves software quality even if the documentation is rarely maintained [47].

Additionally, three independent studies [48–50] indicate that documentation also improves usage. Forward [48] asks software developers about the effectiveness of different attributes of software documentation, and finds that content, maintenance, availability and example usage are the most important attributes. De Souza et al. [49] conduct two surveys, once asking the opinion of maintainers on types of documentation, and once the type of documentation they actually use. They found that source code readability, in-line comments, data model and requirement description are among the important documentation artifacts in both surveys. Dzidek et al. [50] quantitatively assessed the costs and benefits of Unified Modeling Language [51] documentation in a controlled experiment. They found a significant increase in correctness of future changes to software, as well as a significant improvement in software design.

Effective documentation of bioinformatics software and adopting standard code style has specific importance in academia. Much academic software is developed by trainees who soon move on to other employment. These trainees have often had little training in software engineering, which would include the necessity of sufficient documentation [52]. Without good documentation, it becomes difficult to continue developing or using the software. This results in premature abandonment of the software and a waste of the investment in the project. For this reason, documentation can be even more important in academia than in industry, but much academic software remains under-documented.

Peer review of a bioinformatics software paper rarely assesses the software documentation directly. If the reviewers cannot figure out to run the software, however, this may result in rejection of the manuscript. The developer should ensure that described uses of their software remain reproducible. Long after the paper is accepted, published software remains part of developers' résumés and can affect their reputations.

When you lack the time to apply every guideline we propose, you should at least have the following minimum documentation:

1. GitHub or Bitbucket page with code and issue tracker.
2. Readme that covers installation, quick start, input formats and output formats.
3. Reference manual with detailed description of every user-configurable parameter.

The Software Sustainability Institute’s online sustainability evaluation [53] assesses how sustainable and reusable your software is. Many parts of this evaluation focus on adequate documentation. After following our other guidelines, we additionally recommend this evaluation for further detailed suggestions on creating great documentation.

Key Points

- Great bioinformatics software documentation provides detailed instructions for installation, usage and all available options.
- It begins with a quick start guide with walk-through examples.
- Details of software capabilities are navigable through a hierarchical interface.
- Users can request further assistance through a searchable forum.

## Notes

**Mehran Karimzadeh** is a PhD student at the Department of Medical Biophysics, University of Toronto. His research focuses on the dynamics of transcription factor behavior in cancer.

## Notes

**Michael Hoffman** is a Scientist at the Princess Margaret Cancer Centre and Assistant Professor in the Departments of Medical Biophysics and Computer Science, University of Toronto. He researches the application of machine learning techniques to epigenomic data.

## References

[1] Altschul SF, Gish W, Miller W, et al Basic local alignment search tool. J Mol Biol 1990;215:403–10. 2231712 10.1016/S0022-2836(05)80360-2

[2] Kumar S, Nei M, Dudley J, et al MEGA: a biologist-centric software for evolutionary analysis of DNA and protein sequences. Brief Bioinformatics 2008;9:299–306. 18417537 10.1093/bib/bbn017 PMC2562624

[3] Purcell S, Neale B, Todd-Brown K, et al PLINK: a tool set for whole-genome association and population-based linkage analyses. Am J Hum Genet 2007;81:559–75. 17701901 10.1086/519795 PMC1950838

[4] Guex N, Peitsch MC. SWISS-MODEL and the Swiss-PdbViewer: an environment for comparative protein modeling. Electrophoresis 1997;18:2714–23. 9504803 10.1002/elps.1150181505

[5] Li H, Handsaker B, Wysoker A, et al The sequence alignment/map format and SAMtools. Bioinformatics 2009;25:2078–9. 19505943 10.1093/bioinformatics/btp352 PMC2723002

[6] Li H, Durbin R. Fast and accurate short read alignment with Burrows-Wheeler transform. Bioinformatics 2009;25:1754–60. 19451168 10.1093/bioinformatics/btp324 PMC2705234

[7] Rice P, Longden I, Bleasby A. EMBOSS: the European molecular biology open software suite. Trends Genet 2000;16:276–7. 10827456 10.1016/s0168-9525(00)02024-2

[8] Langmead B, Salzberg SL. Fast gapped-read alignment with Bowtie 2. Nat Methods 2012;9:357–9. 22388286 10.1038/nmeth.1923 PMC3322381

[9] Anders S, Huber W. Differential expression analysis for sequence count data. Genome Biol 2010;11:R106. 20979621 10.1186/gb-2010-11-10-r106 PMC3218662

[10] Trapnell C, Williams BA, Pertea G, et al Transcript assembly and quantification by RNA-Seq reveals unannotated transcripts and isoform switching during cell differentiation. Nat Biotechnol 2010;28:511–5. 20436464 10.1038/nbt.1621 PMC3146043

[11] McKenna A, Hanna M, Banks E, et al The Genome Analysis Toolkit: a MapReduce framework for analyzing next-generation DNA sequencing data. Genome Res 2010;20:1297–303. 20644199 10.1101/gr.107524.110 PMC2928508

[12] Smyth GK. limma: Linear models for microarray data In Gentleman R, Carey VJ, Huber W, et al (eds). Bioinformatics and Computational Biology Solutions Using R and Bioconductor. Springer, New York, NY, 2005, 397–420.

[13] Robinson MD, McCarthy DJ, Smyth GK. edgeR: a Bioconductor package for differential expression analysis of digital gene expression data. Bioinformatics 2010;26:139–40. 19910308 10.1093/bioinformatics/btp616 PMC2796818

[14] Zhang Y, Liu T, Meyer CA, et al Model-based analysis of ChIP-Seq (MACS). Genome Biol 2008;9:R137. 18798982 10.1186/gb-2008-9-9-r137 PMC2592715

[15] Quinlan AR, Hall IM. BEDTools: a flexible suite of utilities for comparing genomic features. Bioinformatics 2010;26:841–2. 20110278 10.1093/bioinformatics/btq033 PMC2832824

[16] Sievers F, Wilm A, Dineen AD, et al Fast, scalable generation of high-quality protein multiple sequence alignments using Clustal Omega. Mol Syst Biol 2011;7:539. 21988835 10.1038/msb.2011.75 PMC3261699

[17] Bailey TL, Boden M, Buske FA, et al MEME Suite: tools for motif discovery and searching. Nucleic Acids Res 2009;37:W202–8. 19458158 10.1093/nar/gkp335 PMC2703892

[18] Bolger AM, Lohse M, Usadel B. Trimmomatic: a flexible trimmer for Illumina sequence data. Bioinformatics 2014;30:2114–20. 24695404 10.1093/bioinformatics/btu170 PMC4103590

[19] Dobin A, Davis CA, Schlesinger F, et al STAR: ultrafast universal RNA-seq aligner. Bioinformatics 2013;29:15–21. 23104886 10.1093/bioinformatics/bts635 PMC3530905

[20] Hoffman MM, Buske OJ, Wang J, et al Unsupervised pattern discovery in human chromatin structure through genomic segmentation. Nat Methods 2012;9:473–6. 22426492 10.1038/nmeth.1937 PMC3340533

[21] Huber W, Carey VJ, Gentleman R, et al Orchestrating high-throughput genomic analysis with Bioconductor. Nat Methods 2015;12:115–21. 25633503 10.1038/nmeth.3252 PMC4509590

[22] Picard: a set of command line tools (in Java) for manipulating high-throughput sequencing (HTS) data and formats such as SAM/BAM/CRAM and VCF. http://broadinstitute.github.io/picard/ (31 August 2016, date last accessed).

[23] Doxygen: Generate documentation from source code. http://www.stack.nl/dimitri/doxygen (6 July 2016, date last accessed).

[24] Sphinx: Python documentation generator. http://www.sphinx-doc.org/en/stable/ (6 July 2016, date last accessed).

[25] ReStructuredText. http://docutils.sourceforge.net/rst.html (21 July 2016, date last accessed).

[26] Javadoc—the Java API documentation generator. http://docs.oracle.com/javase/7/docs/technotes/tools/windows/javadoc.html (6 July 2016, date last accessed).

[27] Roxygen: literate programming in R. http://roxygen.org/ (6 July 2016, date last accessed).

[28] Debian Med. https://www.debian.org/devel/debian-med/ (21 July 2016, date last accessed).

[29] Homebrew—the missing package manager for OS X. http://brew.sh/ (6 July 2016, date last accessed).

[30] Comprehensive R Archive Network. https://cran.r-project.org/ (21 July 2016, date last accessed).

[31] PLINK: Whole genome association analysis toolset. http://pngu.mgh.harvard.edu/purcell/plink/ (8 July 2016, date last accessed).

[32] GitHub. https://github.com/ (22 July 2016, date last accessed).

[33] Bitbucket. https://bitbucket.org/ (22 July 2016, date last accessed).

[34] Read the Docs. https://www.readthedocs.org (21 July 2016, date last accessed).

[35] Katz Y, Wang ET, Airoldi EM, et al Analysis and design of RNA sequencing experiments for identifying isoform regulation. Nat Methods 2010;7:1009–15. 21057496 10.1038/nmeth.1528 PMC3037023

[36] Wickham H. ggplot2: Elegant Graphics for Data Analysis. New York: Springer, 2009.

[37] Van Rossum G, Warsaw B, Coghlan N. PEP 8 – Style guide for Python code. https://www.python.org/dev/peps/pep-0008/ (21 July 2016, date last accessed).

[38] Google style guides. https://github.com/google/styleguide (21 July 2016, date last accessed).

[39] Drone: don’t let bugs invade your code. https://drone.io/ (29 July 2016, date last accessed).

[40] Sandve GK, Nekrutenko A, Taylor J, et al Ten simple rules for reproducible computational research. PLoS Comput. Biol 2013;9:e1003285. 24204232 10.1371/journal.pcbi.1003285 PMC3812051

[41] Piccolo SR, Frampton MB. Tools and techniques for computational reproducibility. GigaScience 2016;5:30. 27401684 10.1186/s13742-016-0135-4 PMC4940747

[42] Hastings J, Haug K, Steinbeck C. Ten recommendations for software engineering in research. GigaScience 2014;3:1–4. 25685331 10.1186/2047-217X-3-31 PMC4326482

[43] Artaza H, Chue Hong N, Corpas M, et al Top 10 metrics for life science software good practices. F1000Res 2016;5:2000. 10.12688/f1000research.9206.1 PMC5007752 27635232

[44] Seemann T. Ten recommendations for creating usable bioinformatics command line software. GigaScience 2013;2:1–3. 24225083 10.1186/2047-217X-2-15 PMC4076505

[45] Nguyen-Hoan L, Flint S, Sankaranarayana R. A survey of scientific software development. In: Proceedings of the 2010 ACM-IEEE International Symposium on Empirical Software Engineering and Measurement. New York: Association for Computing Machinery.

[46] Junji Z, Garousi-Yusifolu V, Sun B, et al Cost, benefits and quality of software development documentation: a systematic mapping. J Syst Softw 2015;99:175–98.

[47] Forward A, Lethbridge TC. Software engineering documentation priorities: an industrial study, 2002 http://www.site.uottawa.ca/tcl/gradtheses/aforward/papers/aforwardcascon2002sub.pdf (19 September 2016, date last accessed).

[48] Forward A. Software documentation—building and maintaining artifacts of communication. Master’s thesis, University of Ottawa, Ottawa, ON, Canada.

[49] de Souza SB, Anquetil N, de Oliveira KM. A study of the documentation essential to software maintenance. In: Proceedings of the 23rd Annual International Conference on Design of Communication: Documenting & Designing for Pervasive Information. New York: Association for Computing Machinery.

[50] Dzidek WJ, Arisholm E, Briand LC. A realistic empirical evaluation of the costs and benefits of UML in software maintenance. IEEE Trans Softw Eng 2008;34:407–32.

[51] Booch G, Rumbaugh J, Jacobson I. The Unified Modeling Language User Guide. Reading, MA: Addison-Wesley Professional, 2005.

[52] Dudley JT, Butte AJ. A quick guide for developing effective bioinformatics programming skills. PLoS Comput Biol 2009;5:e1000589. 20041221 10.1371/journal.pcbi.1000589 PMC2791169

[53] Software Sustainability Institute. Online sustainability evaluation. http://www.software.ac.uk/online-sustainability-evaluation (21 July 2016, date last accessed).

[54] Calves B. Reddit post on “What documenation do you expect to accompany bioinformatics programs”. https://www.reddit.com/r/bioinformatics/comments/3x9nfu/what_documentation_do_you_expect_to_accompany/ (7 July 2016, date last accessed).

[55] Hoffman MM. Twitter post on “What bioinformatics software has great documentation?”, 2016a https://twitter.com/michaelhoffman/status/737365309867319296 (7 July 2016, date last accessed).

[56] Hoffman MM. Twitter post on “What do you find helpful in docs?”, 2016b https://twitter.com/michaelhoffman/status/722118783947640833 (7 July 2016, date last accessed).

